# A Test Method for Finding Early Dynamic Fracture of Rock: Using DIC and YOLOv5

**DOI:** 10.3390/s22176320

**Published:** 2022-08-23

**Authors:** Qinghe Zhang, Bing Zhang, Chen Chen, Ling Li, Xiaorui Wang, Bowen Jiang, Tianle Zheng

**Affiliations:** 1 State Key Laboratory Mine Response and Disaster Prevention and Control in Deep Coal Mine, Anhui University of Science and Technology, Huainan 232001, China; 2 School of Civil Engineering and Architecture, Anhui University of Science and Technology, Huainan 232001, China; 3 School of Qilu Transportation, Shandong University, Jinan 250061, China; 4 School of Computer Science and Engineering, Anhui University of Science and Technology, Huainan 232001, China

**Keywords:** fractured rock mass, digital image correlation technology, target detection, YOLOv5, fracture classification and recognition

## Abstract

Intelligent monitoring and early warning of rock mass failure is vital. To realize the early intelligent identification of dynamic fractures in the failure process of complex fractured rocks, 3D printing of the fracture network model was used to produce rock-like specimens containing 20 random joints. An algorithm for the early intelligent identification of dynamic fractures was proposed based on the YOLOv5 deep learning network model and DIC cloud. The results demonstrate an important relationship between the overall strength of the specimen with complex fractures and dynamic fracture propagation, and the overall specimen strength can be judged semi-quantitatively by counting dynamic fracture propagation. Before the initiation of each primary fracture, a strain concentration area appears, which indicates new fracture initiation. The dynamic evolution of primary fractures can be divided into four types: primary fractures, stress concentration areas, new fractures, and cross fractures. The cross fractures have the greatest impact on the overall strength of the specimen. The overall identification accuracy of the four types of fractures identified by the algorithm reached 88%, which shows that the method is fast, accurate, and effective for fracture identification and location, and classification of complex fractured rock masses.

## 1. Introduction

Due to complex geological environments, there are a large number of complex joints and fissures in natural rock masses, and the instability of fractured rock masses is mostly caused by fracture propagation and coalescence [1,2,3,4]. The laws of fracture propagation are very important to the stability of fractured rock masses, and active monitoring is recommended [5,6,7,8]. It has been shown that fracture propagation in rock specimens with complex fractures is complex and changeable during the failure process, and fractures with different positions and sizes have very different effects on the failure of specimens [9]. Under an external load, new fractures can be initiated and propagate at only some of the crack tips, while other fractures appear not to participate in the fracture propagation process [10]. The overall instability of rock specimens with complex fractures is dominated by these dynamic fractures [11,12]. Therefore, it is very important to identify this kind of dynamic fracture quickly and accurately in advance.

Digital image correlation (DIC) is an effective noncontact nondestructive testing process that measures the strain field on a specimen surface; this technology has been widely valued and applied in the field of fractured rock testing [13,14]. Deepanshu et al. quantitatively evaluated the magnitude of tensile and shear damage on the rock surface, based on strain information collected by the DIC technique, quantitatively characterizing that damage initially nucleates at the defect tip, which has a relatively higher degree of tensile and shear damage compared to other areas of the specimen [15]. Wang et al. conducted uniaxial compression tests on rock specimens with prefabricated joints at different angles, and a process of fracture initiation, propagation, and coalescence was observed and analyzed through DIC [16]. The results showed that DIC can record and display precursors of fracture initiation that cannot be captured by ordinary cameras or the human eye, and visually display the strain concentration area on the target material. Zhang et al. used 3D printing technology to prepare rock specimens with fracture networks, and the DIC method to measure the global strain field of specimens, then analyzed the fracture characteristics of fracture network specimens [17,18]. These researchers determined that before the initiation of the primary fracture, a strain concentration area was generated at the crack tip, which more intuitively reflected the fracture law of the complex fracture specimens. This proved again that DIC is an effective method for monitoring fracture initiation and expansion. Although DIC can reliably identify the evolution of fractures, the accurate and efficient capture of dynamic fractures during the failure process is difficult. This is because the dynamic propagation of multiple fractures that are widely distributed in specimens may occur at the same time, making the fractures complex and changeable. Relying only on observations of DIC cloud images by test personnel to identify fractures is not only inefficient but also subjective and inaccurate.

With the rapid development of computer technology and artificial intelligence technology, object detection methods based on deep learning have shown excellent performance in many fields. Combined with a deep neural network and anchor-free feature selection, an end-to-end steel strip surface defect detection model based on You Only Look Once version3 (YOLO-V3) was developed by Kou et al. [19]. By improving the feature extraction and propagation of the detection model, the network characterization ability was enhanced, and the detection accuracy and speed of defect on steel strip surface recognition were improved. An intelligent identification and localization method for detecting wet damage using deep learning and incremental random sampling (IRS) was proposed by Zhang et al. [20]; this method exhibited good performance and advantages in detecting and locating wet damage in asphalt pavements. Asphalt pavement damage was predicted through the deep learning methods by Hamed et al. [21], and nine types of pavement damage were automatically detected, classified, and segmented by using Google Street View images and a deep learning framework. High-level semantic information from original images could also be automatically extracted by deep learning for intelligent fracture recognition in concrete or rock materials, providing a new way to intelligently identify fractures in concrete or rock-like materials. Ju proposed a pavement fracture depth network architecture to detect sealed and unsealed fractures with complex road backgrounds, and the performance of three intelligent detection algorithms was compared, namely, fracture DN, faster region-based convolutional neural network (RCNN), and single-shot multibox detection with 300 × 300 input resolution (SSD300) [22]. Intelligent detection technology to classify, identify, and quantify concrete damage has been used by scholars such as Cui [23], Jiang [24], and Wei [25]. Song et al. applied deep learning technology combined with structured light technology to detect and quantify fractures on the surface of concrete structures [26]. In terms of the selection of the deep convolutional neural network algorithm, the YOLOv5 algorithm is a relatively new framework of the YOLO family, whose model for intelligent detection of targets has been used by many researchers. Yan et al. combined multispectral imaging technology and target detection, and added the scSE module to CSPDarknet and CSP modules according to the model structure of YOLOv5. An intelligent classification method for coal gangue has been proposed [27]. Studies have shown that the YOLOv5 algorithm model is faster and more accurate than the traditional model and can effectively improve the accuracy of target recognition [28,29,30,31].

In summary, the combination of DIC, which is very sensitive to the initiation and propagation of early microfractures in the failure process of rocks with complex fissures, and the YOLOv5 algorithm, which is an intelligent detection and identification algorithm with fast detection and high accuracy, may be utilized for the intelligent identification of rock failure with complex fractures. Therefore, in this paper, a failure test was designed for a rock specimen with 20 random fractures. The whole process of the specimen’s deformation and failure was collected in real time by DIC, and the evolution cloud map of the strain field was used as a training dataset. The evolution of dynamic fractures in the specimen was identified automatically and intelligently by the YOLOv5 algorithm, which was used to judge the overall failure of the specimen.

## 2. Principle and Development of YOLOv5

**Figure 1:**
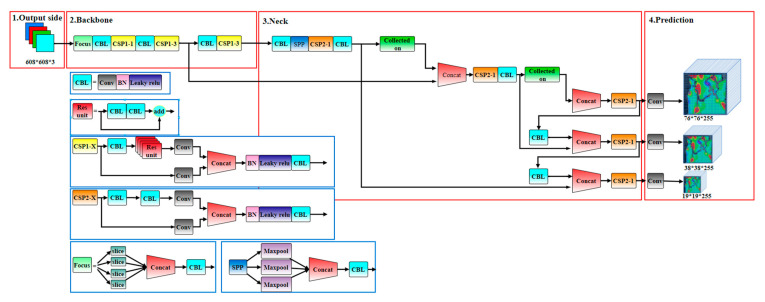
Structure of YOLOv5 algorithm model.

YOLO, an object recognition and localization algorithm based on a deep neural network, with an inherent speed advantage, can be used in real-time systems. The YOLO algorithm regards the target detection task as a single regression prediction problem and obtains bounding frame coordinates and class probabilities directly from image pixels. It uses the combined convolutional neural network to extract the multiscale features of the image, then fuses the features through the fully connected network layer, transmits the image features to the prediction layer, processes the network prediction results, and predicts the image features, finally generating the target bounding frame and the prediction class probability. The YOLOv5 algorithm has the strongest reasoning speed and the lightest model size in the YOLO target detection series; its model structure is shown in Figure 1.

**Figure 2:**
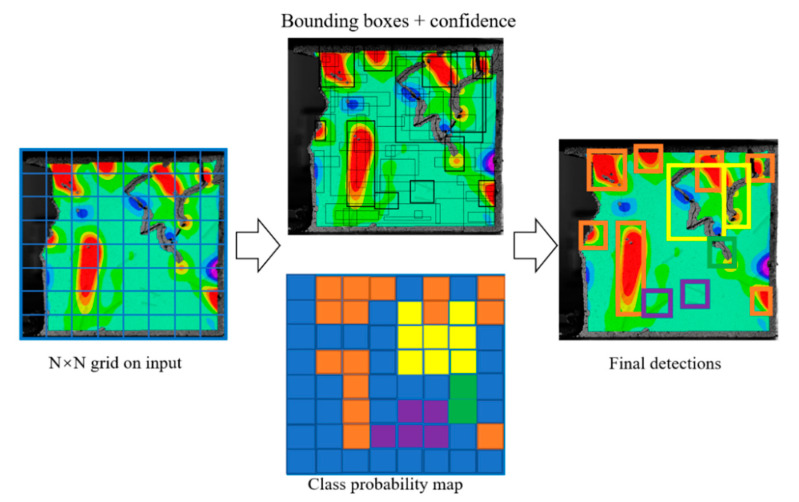
Dynamic fracture detection and identification of specimens.

In this paper, a new detection model is proposed based on the YOLOv5 framework; the intelligent detection process for dynamic fractures is shown in Figure 2.

(1) The input fracture network graph was divided into *S* × *S* grids. Each grid generated a prior frame for targets of different scales, responsible for tracking and recognizing the target when the centre of the target was located in a certain grid. The confidence degree *c* was used to represent the probability of target classification and the performance of target matching in the prior frame [32]:

(1) c=Pr(object)×IOU(pred,truth)

where *Pr (object)* is the probability of the object in the prediction frame. If there is no target in the prediction frame, it is 0; otherwise, it is 1. *IOU (pred, truth)* is the intersection ratio of the prediction frame and the truth frame.

(2) The divided fracture images were normalized, and the normalized fracture dataset was sent to the underlying feature extraction network for feature extraction.
(3) The prediction frame was divided into different sizes. For different detection targets, the position of the prediction frame, namely, the coordinate of the central point, was calculated.
(4) According to the offset value of the predicted coordinates, the target center point position and the width and height of the prediction frame were calculated.
(5) The target identification results were output.

## 3. Failure Test of the Fractured Rock Specimen

### 3.1. Preparation of Rock Specimens with Complex Fractures

**Figure 3:**
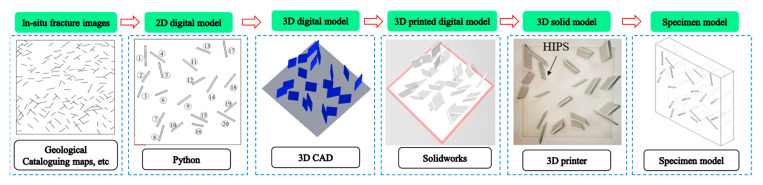
Principle and process of specimen production.

The test samples with complex fractures were produced by 3D printing technology, as shown in Figure 3. This method is an emerging technology developed in recent years that solves the problem of specimen preparation for physical models of fracture networks [33]. The basic process of the method was as follows: (1) the fracture network mould was 3D printed with water-soluble material; (2) the specimen was cast using high-strength cement mortar and a fracture network mould; and (3) after the specimen was cured, it was placed into clean water, and the water-soluble material and dissolved to leave rock specimens with an open fracture network.

#### 3.1.1. 3D Printing of the Fracture Network Model

In practical engineering, a certain statistical law is usually obeyed by the distribution and size of fractures in rock masses with complex fracture networks. Therefore, in this paper, the rock mass of the dam foundation of a hydropower station in the upper reaches of the Lancang River in southwest China was taken as the research prototype [34], and a Python program for random fracture generation was compiled based on the Monte Carlo principle. Using this program, 20 randomly distributed fractures were generated in the range of 100 × 100 mm. Among them, the normal distribution exhibited a fracture width between 0.8 and 1 mm and a fracture length between 10 and 20 mm. After generation, the 2D digital model was imported into computer-aided design (CAD) and Solidworks software, and the 3D digital model was generated using the plane stretching command to stretch 20 fractures 20 mm in the positive direction of the Z axis. The 3D printed digital model was imported into the 3D printer for fracture printing. The printing material was polyvinyl alcohol (PVA), and the printing method was the melting accumulation method with an accuracy of 0.1 mm. The above process is shown in Figure 3.

#### 3.1.2. Preparation of Similar Rock Material Specimens for the Fracture Network

Cement mortar, as a kind of rock material, has the advantages of controllable composition and good mechanical properties, so cement mortar material was chosen to simulate real rock in this paper. The material parameters of the cement mortar are shown in Table 1 below.

**Figure 4:**
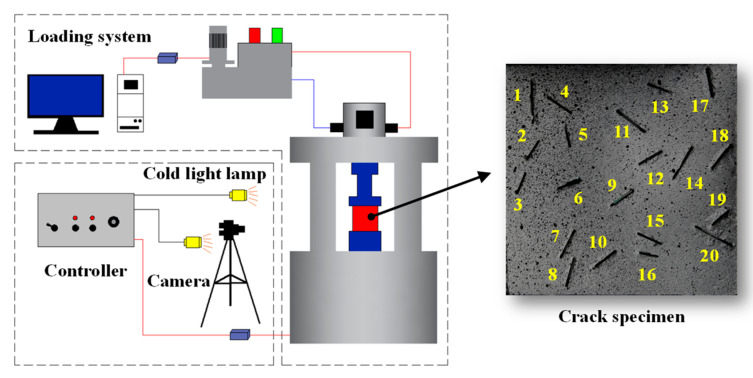
Diagram of the test system.

The sample of rock-like material in the fracture network is shown in the right figure of Figure 4. The preparation method was as follows:

(1) The specimen mould was selected with an internal size of 100 × 100 × 20 mm (length × width × height), and the 3D fracture solid model was placed in the mould. Cement mortar, with a material ratio of ordinary Portland cement:sand:water = 1:1:0.4, was poured into the mould, and full oscillation was achieved.
(2) The mould was removed after 27 h, and the specimen was placed in a standard curing room for 28 days.
(3) After curing, the specimen was immersed in clean water for 48 h, and the PVA material was dissolved, forming a fracture network.
(4) The specimen was dried naturally at room temperature and polished smoothly with sandpaper. White paint and black paint were sprayed successively on one side of the specimen to form a speckled field. The white paint was the color base, and the black paint particles naturally fell on the white primer, which conveniently allowed the DIC equipment to collect and calculate the strain field.

### 3.2. Test Instrument

The test system used in the tests is shown in Figure 4. The loading equipment was a Servo universal testing machine, and the displacement load was applied to the specimen at a load rate of 0.3 mm/min. The VIC-2DTMSystem was used in the DIC unit, and a binocular industrial camera with a resolution of 2560 × 1920 pixels and a shooting rate of 10 frames/s was employed to capture the changes in the speckled field on the specimen surface during real-time loading. During the shooting, light-emitting diode (LED) cold light was used to fill the light on the specimen surface so that the images of speckles recorded by the camera exhibited high definition. Before loading the specimen, its upper and lower ends were coated with Vaseline to reduce the adverse effect of end friction on the test.

## 4. Test Results and Analysis

### 4.1. Mechanical Properties of the Specimens

**Figure 5:**
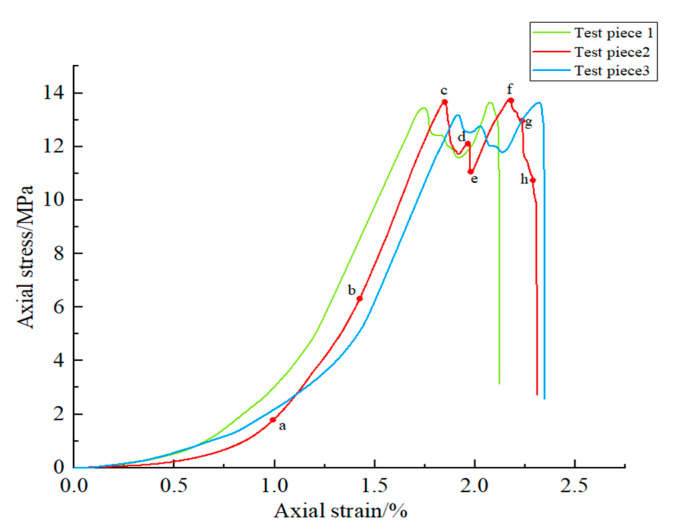
Stress-strain curve.

As shown in Figure 5, the peak strength and elastic modulus of the three specimens were basically similar, indicating that the specimens prepared by 3D printing technology had stable mechanical properties and good repeatability.

The deformation and fracture characteristics of each specimen were roughly similar, and specimen #2 was selected for analysis in this paper. Table 2 shows the fracture propagation at the time corresponding to the marked points a–h on the stress–strain curve. In addition, digital images collected throughout the test were imported into VIC-2D to calculate the strain field during the loading process. The specific processing process is shown below.

(1) All the pictures captured during the loading damage to the specimen were extracted from the camera. Based on the duration of the test piece damage phase, the feature pictures were taken as the observation objects.
(2) The feature pictures were imported into the digital image correlation calculation software (Vic-2D), and the strain field cloud map and strain field data information obtained by digital image correlation calculation, where each picture had a set of strain field data information, all saved in Excel format.
(3) The strain field information from the separate Excel tables was summarized into one table using a Python program, to obtain the strain field variation with time order.

As seen from Figure 5 and Table 2, the deformation and fracture process of the specimen with a complex fracture distribution can be roughly divided into the following four stages:

(1) In the initial compaction stage (0–a), similar to the real rock specimen, with significant initial nonlinear deformation characteristics, the original microdefects inside the specimen are gradually compacted. At this time, the precast fractures in the specimen did not propagate, and the strain field of the specimen was relatively uniform.
(2) In the approximate linear stage (a–c), the stress–strain curve increased approximately linearly with increasing load. At point b, strain concentration occurred at the No. 17 fracture, but it had little effect on the overall strength of the specimen.
(3) In the fracture initiation and propagation stage (c–f), when the stress increased to point c, strain concentration and fracture propagation occurred at the No. 2, 3, 11, 12, 14, and 17 fractures of the specimen. After c and before f, the stress–strain curve was accompanied by multiple stress drops and stress redistribution. In this process, although some new fractures were generated in the specimen, no surface-penetrating fracture appeared.
(4) In the postpeak stage (f–h), when the stress reached the peak stress point f, the new fracture and the original fracture were connected in the specimen, and the bearing capacity of the specimen decreased rapidly, resulting in brittle failure. This indicates that the failure process of the fracture network specimens incorporated the germination and expansion of new fractures, and the bonding and coalescence of primary fractures.

### 4.2. Local Strain Response of Dynamic Fractures

**Figure 6:**
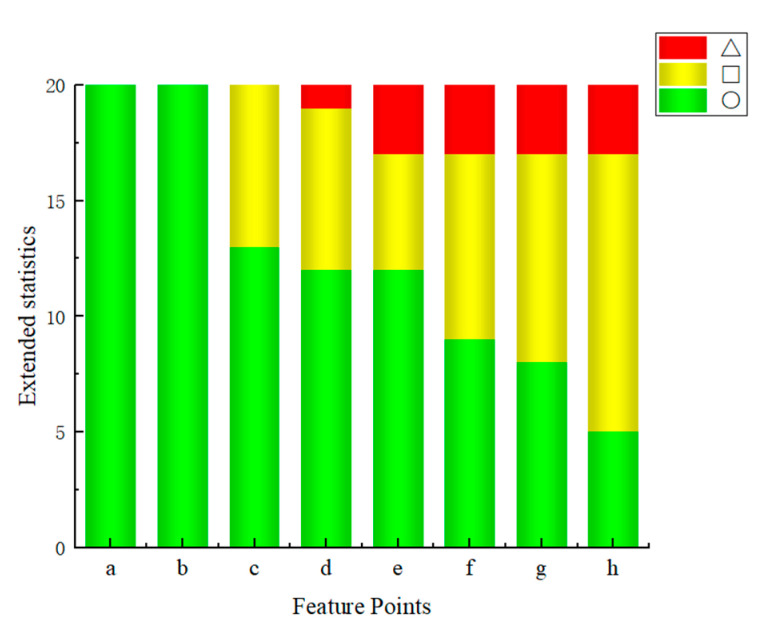
Extended statistics.

To further reveal the influence of single fracture expansion on the overall strength of the specimen, the development and expansion of 20 fractures were analyzed in detail, as shown in Table 3 and Figure 6.

It can be found that a single fracture had an important effect on the overall strength of the specimen.

(1) In the initial compaction stage, the strain field of the specimen was relatively uniform, and only a small number of primary crack tips showed strain concentrations, which were not obvious and did not affect the overall basic strength of the specimen.
(2) In the approximate linear stage, at point c, the ends of the No. 2, 3, 11, 12, 14, and 17 prefabricated fractures began to generate new fractures, and a dark red strain concentration area covering the fractures appeared in the strain field cloud map. At the same time, the elastic stage came to an end, and multiple fractures were initiated simultaneously.
(3) In the fracture initiation and propagation stage, at point d, the No. 1, 2 and 3 prefabricated fractures on the left side of the specimen began to overlap with each other, and the left side was close to detaching. At the same time, the anti-wing fracture sprouting at the upper end of the No. 11 fracture was observed to overlap with that of the No. 13 fracture, and the wing fracture at the lower end of the No. 11 fracture overlapped with the anti-wing fractures at the upper ends of the No. 12 fractures. There was overlap between the reverse wing fractures generated at the upper end of No. 12 and the lower end of No. 14, and the wing fractures generated at the lower end of No. 17 and the upper end of No. 14 also overlapped. The positions of the above fractures in the strain field cloud map demonstrated significant changes. When the fracture propagation was significant, the strain data of the new fractures were lost, and the strain cloud map shows no colour. At the same time, the stress–strain curve was observed to show a small amplitude stress fluctuation.
(4) In the postpeak stage, at point f, a wing fracture extending upwards germinated at the upper end of fracture No. 4, and an inclined anti-wing fracture at the lower end of fracture No. 6 overlapped with a wing fracture at the upper end of fracture No. 7. At point g, the fractures developed continuously, the new wing fractures at the top of fracture No. 4 developing to the edge of the specimen, inclined reverse wing fractures germinating at the top of fractures No. 6 and No. 7. Inclined secondary fractures germinated in the middle of fracture No. 13, and the tensile wing fractures at the bottom of fracture No. 14 propagated along the axis, resulting in a stress drop of 1.14 MPa in the stress–strain curve. At point h, the wing fracture generated at the upper end of fracture No. 9 overlapped with the reverse wing fracture generated at the lower end of fracture No. 12, and the prefabricated fractures 4, 5, 6, 7, 8, 9, 11, 12, 13, 14, and 17 overlapped with each other to form a complex fracture network, resulting in a stress drop and eventually forming a macroscopic penetrating fracture leading to failure of the specimen.

In Figure 6, it can be seen from the statistical analysis of single fracture propagation, strength law of specimens, and the DIC cloud map that the characteristics of fracture propagation and failure of specimens can be clearly traced by using the evolution law of the specimen strain field. When loading occurred, before the initiation of each primary fracture, the strain concentration area always appeared first, which indicates the initiation of new fractures. At the early stage of fracture initiation and propagation, multiple fractures may propagate, and simultaneous fracture initiation occurs. At the late stage of fracture propagation, the stress fluctuated slightly, and the fracture propagation was very complicated. In the postpeak stage, the fissure germination became more dynamic, and the expansion more complex. All these factors bring great challenges to fracture monitoring. In addition, at the end of the elastic stage the specimen with a complex fracture distribution was at approximately 97% of peak stress, and the specimen rapidly failed after reaching peak stress. Therefore, it is very important to consider the bearing capacity of specimens based on fracture propagation.

## 5. Intelligent Detection of Dynamic Fractures

### 5.1. Experimental Treatment and Results

#### 5.1.1. Software and Hardware Conditions

A computer with an AMD Ryzen 9 3900X processor, 3.80 GHz main frequency, 32 GB memory, a GeForceGTX 2080Ti graphics card, 12 GB video memory, Windows 10, 64-bit operating system was used. The YOLOv5 algorithm model was built using Python and PyTorch.

#### 5.1.2. Dataset

(1) Dataset preprocessing

**Figure 7:**
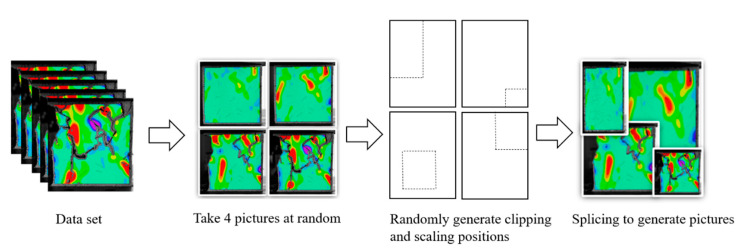
Mosaic data enhancement principle.

The deep learning dataset was established based on the strain field evolution cloud map obtained from the failure testing of the complex fracture specimens. A total of 1000 strain field cloud images were collected, all of which were taken as sample sets. Eighty percent of the sample sets were training sets, and the remainder were test sets. To enrich the dataset, mosaic data enhancement technology was used in this study at the input, as shown in Figure 7. Through random zooming, random clipping, and random arrangement for stitching supplements, many target object sample data were enhanced. Mosaic data enhancement technology makes the target object smaller, which eventually makes the detection of the target object more accurate to improve the robustness of the network. This can effectively solve the problem of insufficient small target objects in the dynamic fracture detection sample.

(2) Fracture classification

**Figure 8:**
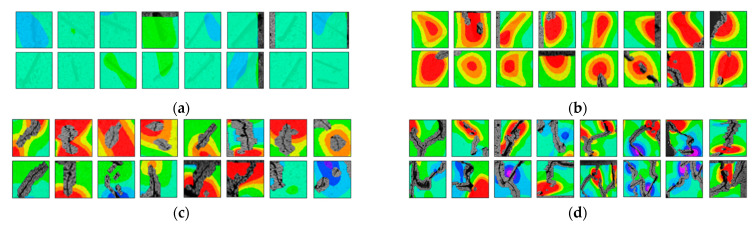
Fracture classification. (**a**) Original Fissures (OF); (**b**) Strain Concentration Areas (SCA); (**c**) Emerging Fissures (EF); (**d**) Cross Fissure (CF).

The test results showed that the initial initiation of a fracture greatly affected the strength of the whole rock mass. Synchronous monitoring of all fractures and the capture of dynamic fractures are the main problems to be solved in this paper. Therefore, the fractures at different stages were divided into four types: primary fractures (OF), strain concentration area (SCA), new fractures (EF), and cross fractures (CF). The four types are shown in Figure 8. Figure 8a shows prefabricated primary fractures in the specimen. Figure 8b shows the area of strain concentration on the specimen during the loading process, which was the precursor of new fracture initiation. Figure 8c shows a newly formed fracture at the tip of the primary fracture; Figure 8d shows a cross fracture that occurred when the new fracture expanded and overlapped with the primary fracture.

#### 5.1.3. Test Results

**Figure 9:**
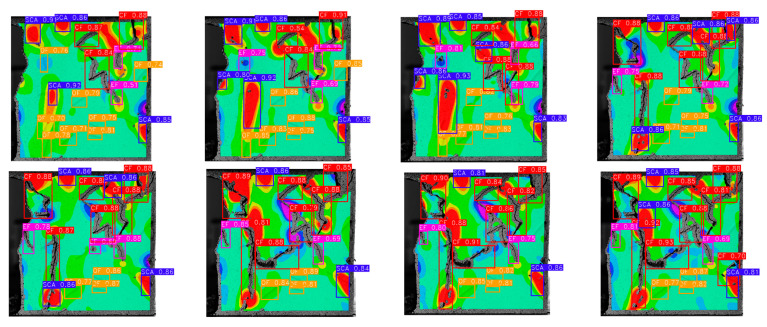
Dynamic fracture detection results during specimen failure.

The dataset was used for 500 iterations of training. In the training process, the random gradient descent algorithm was used, the batch size was 64, the momentum was 0.937, the attenuation was 0.0005, and the learning rate was initialized to 0.01. After the training, the images of the test set were input into the trained model. Then, the prediction frame and confidence degree of the target were output by the neural network algorithm, and the detection results for dynamic fractures were obtained, as shown in Figure 9.

### 5.2. Algorithm Evaluation

#### 5.2.1. Measurement Index of Model Recognition Accuracy

The model accuracy was measured by *F*_1_ and *mAP* [35,36]. The measurement indexes depend on the calculation of accuracy and recall rate.

The accuracy rate *P* is the ratio of all positive samples that are actually true, which measures the recognition ability of the model with regard to negative samples; the recall rate R is the accuracy rate of each classification, which measures the recognition ability of the model with regard to positive samples. The calculation formula is shown in Equations (2) and (3) [37]:

(2) $$P=\frac{TP}{TP+FP}$$

(3) $$R=\frac{TP}{TP+FN}$$

where *P* is the accuracy rate, *R* is the recall rate, *TP* is the number of positive samples predicted by the model, *FP* is the number of negative samples predicted by the model to be positive, and *FN* is the number of positive samples predicted by the model to be negative.

*F*_1_ can avoid extreme situations where the accuracy rate and recall rate differ greatly, and can better reflect the whole. The calculation formula is shown in Equation (4):

(4) $$F1=2\frac{P\cdot R}{P+R}$$

where *F*_1_ is a comprehensive index combining the precision rate and recall rate.

For a certain class of samples with N positive examples, all test results are arranged in descending order according to the confidence degree. Each additional positive example corresponds to an accuracy value, and the average accuracy of the class can be obtained by taking its average value. The calculation formula is shown in Equation (5):

(5) $$AP=\frac{1}{n}\sum_{i=1}^{n}P_{i}=\frac{1}{n}P_{1}+\frac{1}{n}P_{2}+\cdots +\frac{1}{n}P_{n}$$

where *n* is the number of samples, *AP* is the average accuracy, and *Pi* is the accuracy value of a positive example.

*mAP* is the mean of the average accuracy of all classes, which measures the overall trend of model accuracy and recall rate. The higher the *mAP*, the easier it is for the model to maintain high accuracy at high recall rates. When the threshold value of the *IOU* of the confusion matrix is 0.5, the mean value of the average accuracy of all classes is marked as *mAP*@0.5. The calculation is shown in Equation (6):

(6) $$mAP=\frac{1}{C}\sum_{k=1}^{C}AP_{k}$$

where *mAP* is the mean of the average accuracy, *C* is the number of target detection categories, and *AP_k_* is the average accuracy of target detection for a certain category.

#### 5.2.2. Evaluation Results

**Figure 10:**
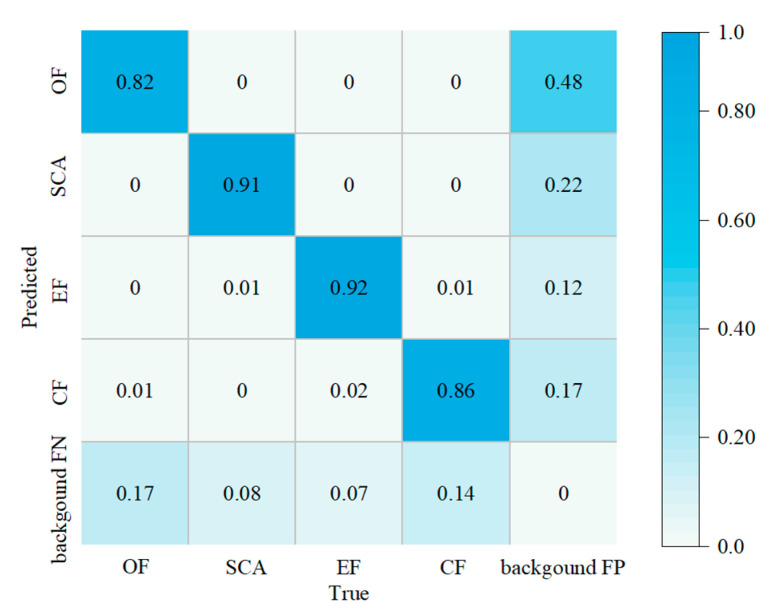
Identification model confusion matrix.

The confusion matrix obtained from network model training is shown in Figure 10. Figure 10 shows a confusion matrix in which each column represents the category of predictions, the total number and value of each column are expressed as the number of data and the number of actual data predicted for that category. Each row represents the real category of data, and the total amount of data in each row represents the number of data instances of this category. It indicates good stability in detecting various types of fractures.

**Figure 11:**
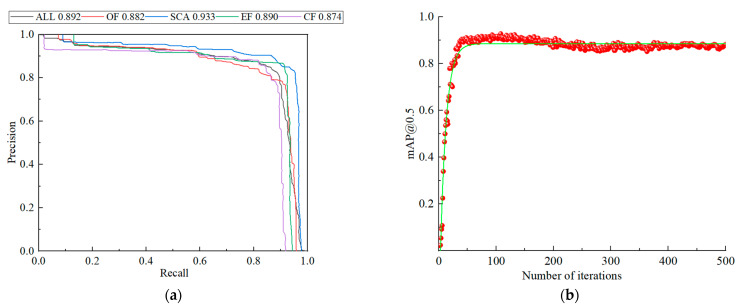
Model training curve. (**a**) P-R curve; (**b**) mAP@0.5.

Based on the constructed intelligent recognition algorithm for dynamic fractures, the accuracy–recall curve and *mAP* curve were obtained. Figure 11a shows the P–R curve, a 2D curve with accuracy rate and recall rate as the vertical and horizontal coordinates. The area enclosed by the curve is the *AP*. The *APs* corresponding to the four types were 88.2%, 92.3%, 89.0%, and 87.4%. The overall *AP* was 89.2%; the average accuracy of the model was relatively high, and the performance of the random model was better. In Figure 11b, the value of *mAP*@0.5, which measures the overall performance of the network, is shown to be above 80%, reaching 92%, indicating that the model had a strong high ability and the algorithm had a good detection effect.

To verify the identification effect of the model, the test sample set was used for training, and the *F*_1_ scores were obtained, as shown in Table 4. The *F*_1_ corresponding to the four types was 85%, 91%, 89%, and 87%. In a global statistical sense, when the average *F*_1_ of the recognition of the four types of fractures reached 88%, it was proven that the algorithm could accurately and intelligently identify the four types of dynamic fractures, to verify the feasibility and accuracy of the target detection model.

## 6. Conclusions

(1) The failure process of specimens with complex fractures is often accompanied by the propagation and coalescence of multiple dynamic fractures. DIC monitoring showed that before the initiation of each primary fracture, the strain concentration area always appeared first, which indicated the initiation of new fractures.
(2) There was an important relationship between the overall strength of the specimen and the propagation of a single fracture. At the end of the elastic deformation stage, the stress was close to its peak, at approximately 97% of peak stress. In the fracture propagation stage, there were many stress fluctuations, which had an important relationship with the cross fractures. After reaching peak stress, the specimen was rapidly destroyed. The overall strength of the specimen can be evaluated semiquantitatively by the statistics of the expansion of each fracture.
(3) Based on the strength law and strain field cloud map of specimens, the dynamic evolution of primary fractures can be divided into four types: primary fractures, stress concentration area, new fractures, and cross fractures, among which the cross fractures had the greatest impact on the overall strength of the specimen.
(4) Based on YOLOv5, an intelligent detection algorithm for identifying dynamic fractures in rock specimens with complex fractures was established. The detection results show that the value of *mAP*@0.5 was above 80%, reaching 91%. This model can be used for intelligent and accurate identification of dynamic fractures, and the state of each fracture can be determined intelligently based on the type of fracture.
(5) The established intelligent detection model of dynamic fractures demonstrated fast detection speed and high accuracy. The *F*_1_ corresponding to the four fracture types was 85%, 91%, 89%, and 87%, and the overall identification accuracy reached 86%.
(6) In underground coal mine engineering, crack expansion and penetration may occur until rupture channels appear, for various reasons such as cyclic mining and gas seepage surges, with potential for disaster. This paper proposes a combination of the YOLOv5 deep learning network model and DIC technology, for the intelligent detection and identification of crack extensions in complex rock specimens. The experimental evaluation has shown that the method is fast, accurate, and effective in the identification, localization, and classification of cracks in complex fractured rock masses. Therefore, it is proposed that intelligent monitoring of rock damage can be realized to provide improved damage warning and directions for engineering safety protection.

## Figures and Tables

**Table 1:** Material parameters of cement mortar.

Material Type	Density ρ/g·cm^−3^	Uniaxial Compressive Strength σc/MPa	Elastic Modulus E/GPa	Poisson’s Ratio μ	Tensile Strength σt/MPa
Mortar	2.04	80.26	11.2	0.18	5.42

**Table 2:** Specimen failure and strain field evolution.

Points	Fissure Development	Strain Fields	Points	Fissure Development	Strain Fields
a	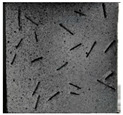	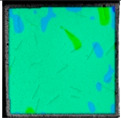	b	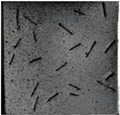	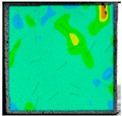
c	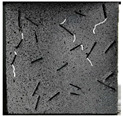	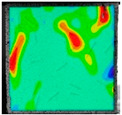	d	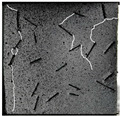	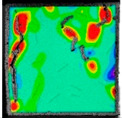
e	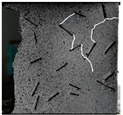	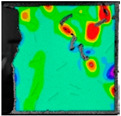	f	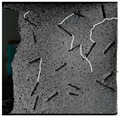	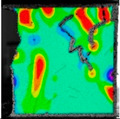
g	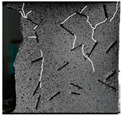	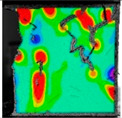	h	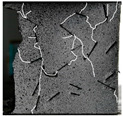	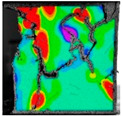

**Table 3:** Statistics of fracture propagation.

No.	1	2	3	4	5	6	7	8	9	10	11	12	13	14	15	16	17	18	19	20
a	○	○	○	○	○	○	○	○	○	○	○	○	○	○	○	○	○	○	○	○
b	○	○	○	○	○	○	○	○	○	○	○	○	○	○	○	○	○	○	○	○
c	○	□	□	○	○	○	○	○	○	○	□	□	○	□	○	○	□	○	○	○
d	□	□	△	○	○	○	○	○	○	○	□	□	□	□	○	○	□	○	○	○
e	△	△	△	○	○	○	○	○	○	○	□	□	□	□	○	○	□	○	○	○
f	△	△	△	□	○	□	□	○	○	○	□	□	□	□	○	○	□	○	○	○
g	△	△	△	□	□	□	□	○	○	○	□	□	□	□	○	○	□	○	○	○
h	△	△	△	□	□	□	□	□	□	○	□	□	□	□	○	○	□	○	○	□

Note: “○” means no new fractures generated around the precast fracture, “□” means new fractures generated around the precast fracture, and “△” means that the new fractures around the precast fracture expanded significantly.

**Table 4:** F1 scores.

Type	All	OF	SCA	EF	CF
F1	0.88	0.85	0.91	0.89	0.87

## Data Availability

Not applicable.

## References

[1] Chen W., Li S., Zhu W., Qiu X. (2003). Experimental and numerical research on crack propagation rock under compression. Chin. J. Rock Mech. Eng..

[2] Liu X., Zhu W., Zhang P., Li L. (2021). Failure in rock with intersecting rough joints under uniaxial compression. Int. J. Rock Mech. Min. Sci..

[3] Sivakumar G., Maji V.B. (2021). Crack Growth in Rocks with Preexisting Narrow Flaws under Uniaxial Compression. Int. J. Geomech..

[4] Lin H., Sheng B. (2021). Failure Characteristics of Complicated Random Jointed Rock Mass Under Compressive-Shear Loading. Geotech. Geol. Eng..

[5] Wang H., Zhang B., Yuan L., Wang S., Yu G., Liu Z. (2022). Analysis of precursor information for coal and gas outbursts induced by roadway tunneling: A simulation test study for the whole process. Tunn. Undergr. Space Technol..

[6] Miao S., Pan P.Z., Wu Z., Li S., Zhao S. (2018). Fracture analysis of sandstone with a single filled flaw under uniaxial compression. Eng. Fract. Mech..

[7] Jin Z., Johnson S., Fan Z. (2010). Subcritical propagation and coalescence of oil-filled cracks: Getting the oil out of low-permeability source rocks. Geophys. Res. Lett..

[8] Fan Z., Jin Z., Johnson S. (2014). Oil-Gas Transformation Induced Subcritical Crack Propagation and Coalescence in Petroleum Source Rocks. Int. J. Fract..

[9] Wang X., Wang E., Liu X., Zhou X. (2021). Failure mechanism of fractured rock and associated acoustic behaviors under different loading rates. Eng. Fract. Mech..

[10] Ma G., Li M., Wang H., Chen Y. (2020). Equivalent discrete fracture network method for numerical estimation of deformability in complexly fractured rock masses. Eng. Geol..

[11] Luo K., Zhao G., Zeng J., Zhang X., Pu C. (2018). Fracture experiments and numerical simulation of cracked body in rock-like materials affected by loading rate. Chin. J. Rock Mech. Eng..

[12] Suzuki A., Watanabe N., Li K., Horne R.N. (2017). Fracture network created by 3-D printer and its validation using CT images. Water Resour. Res..

[13] Munoz H., Taheri A. (2017). Specimen aspect ratio and progressive field strain development of sandstone under uniaxial compression by three-dimensional digital image correlation. J. Rock Mech. Geotech. Eng..

[14] Cao R., Lin H., Cao P. (2018). Strength and failure characteristics of brittle jointed rock-like specimens under uniaxial compression: Digital speckle technology and a particle mechanics approach. Int. J. Min. Sci. Technol..

[15] Deepanshu S., Ahmadreza H., Gabriel W. (2021). Damage monitoring in rock specimens with pre-existing flaws by non-linear ultrasonic waves and digital image correlation. Int. J. Rock Mech. Min. Sci..

[16] Wang B., Jin A., Sun H., Wang S. (2021). Study on the rupture mechanism of 3D printed rough cross-sectional specimens containing different angles based on DIC. Rock Soil Mech..

[17] Zhang K., Qi F.F., Chen Y.L. (2020). Deformation and fracturing characteristics of fracture network model and influence of filling based on 3D printing and DIC technologies. Rock Soil Mech..

[18] Zhang K., Zhang K. (2021). Gray and texture features of strain field for fractured sandstone during failure process. J. China Coal Soc..

[19] Kou X., Liu S., Cheng K., Qian Y. (2021). Development of a YOLO-V3-based model for detecting defects on steel strip surface. Measurement.

[20] Zhang J., Yang X., Li W., Zhang S., Jia Y. (2020). Automatic detection of moisture damages in asphalt pavements from GPR data with deep CNN and IRS method. Autom. Constr..

[21] Hamed M., Yaw A.G., William G.B. (2020). Deep machine learning approach to develop a new asphalt pavement condition index. Constr. Build. Mater..

[22] Ju H., Li W., Tighe S., Zhai J., Xu Z., Chen Y. (2019). Detection of sealed and unsealed cracks with complex backgrounds using deep convolutional neural network. Autom. Constr..

[23] Cui X., Wang Q., Dai J., Zhang R., Li S. (2021). Intelligent recognition of erosion damage to concrete based on improved YOLO-v3. Mater. Lett..

[24] Jiang Y., Pang D., Li C. (2021). A deep learning approach for fast detection and classification of concrete damage. Autom. Constr..

[25] Wei F., Yao G., Yang Y., Sun Y. (2019). Instance-level recognition and quantification for concrete surface bughole based on deep learning. Autom. Constr..

[26] Song Ee P., Seung-Hyun E., Haemin J. (2020). Concrete crack detection and quantification using deep learning and structured light. Constr. Build. Mater..

[27] Yan P., Sun Q., Yin N., Hua L., Shang S., Zhang C. (2022). Detection of coal and gangue based on improved YOLOv5.1 which embedded scSE module. Measurement.

[28] Tan Y., Cai R., Li J., Chen P., Wang M. (2021). Automatic detection of sewer defects based on improved you only look once algorithm. Autom. Constr..

[29] Haneul C., Chai Y.U., Kyungmo K., Hyungkeun K., Taeyeon K. (2021). Application of vision-based occupancy counting method using deep learning and performance analysis. Energy Build..

[30] Wu L., Zhang L., Shi J., Zhang Y., Wan J. (2022). Damage detection of grotto murals based on lightweight neural network. Comput. Electr. Eng..

[31] Shihavuddin A.S.M., Rashid M.R.A., Maruf M.H., Hasan M.A., ul Haq M.A., Ashique R.H., Al Mansur A. (2021). Image based surface damage detection of renewable energy installations using a unified deep learning approach. Energy Rep..

[32] Li S., Gu X., Xu X., Xu D., Zhang T., Liu Z., Dong Q. (2021). Detection of concealed cracks from ground penetrating radar images based on deep learning algorithm. Constr. Build. Mater..

[33] Liu Q.S., He F., Deng P.H., Tian Y.C. (2019). Application of 3D printing technology in physical modelling in rock mechanics. Rock Soil Mech..

[34] Deng S., Wang X., Yu J., Zhang Y., Liu Z., Zhu Y. (2018). Simulation of Grouting Process in Rock Masses Under a Dam Foundation Characterized by a 3D Fracture Network. Rock Mech. Rock Eng..

[35] Li R., Hu X., Chen F., Wang X., Xiong H., Wu H. (2022). A systematic framework for DEM study of realistic gravel-sand mixture from particle recognition to macro- and micro-mechanical analysis. Transp. Geotech..

[36] Song K., Jung J.Y., Lee S.H., Park S. (2021). A comparative study of deep learning-based network model and conventional method to assess beach debris standing-stock. Mar. Pollut. Bull..

[37] Zhang F., Ren F., Li J., Zhang X. (2022). Automatic stomata recognition and measurement based on improved YOLO deep learning model and entropy rate superpixel algorithm. Ecol. Inform..

